# Tirofiban Benefits the Outcome of Stroke Patients With Large Artery Atherosclerosis Achieving Full Reperfusion and High NIHSS Scores

**DOI:** 10.1111/ene.70070

**Published:** 2025-02-04

**Authors:** Adrià Arboix, Olga Parra

**Affiliations:** ^1^ Department of Neurology, Hospital Universitari Sagrat Cor Universitat de Barcelona Barcelona Catalonia Spain; ^2^ Department of Pneumology, Hospital Universitari Sagrat Cor Universitat de Barcelona Barcelona Catalonia Spain

1

The current intra‐arterial reperfusion therapies allow high rates of recanalization and favorable clinical outcomes with low complication rates, but sufficient reperfusion following endovascular therapies is unsuccessful in about 30% of stroke patients [1]. Platelet aggregation stimulated by mechanical thrombectomy instruments may be a contributing factor for reperfusion failure. Tirofiban—a selective and rapidly activated antagonist of the platelets non‐peptide glycoprotein IIb/IIIa receptors—can reversibly suppress platelet aggregation and may inhibit platelet‐mediated thrombus formation in acute stroke [2].

In clinical practice, however, experience with the use of intravenous tirofiban regarding efficacy and safety in ischemic stroke patients have yielded conflicting results. In a systematic review and meta‐analysis of seven randomized controlled trials (RCTs) with 2088 stroke patients, tirofiban as compared with control of regular treatment regimen or endovascular thromboembolectomy, was associated with a significant increase in the number of patients with Modified Rankin Scale (mRS) = 0 (no symptoms at all) after 90 days and a significant decrease in National Institutes of Health Stroke Scale (NIHSS) score after 7 days [3]. Regarding safety outcomes, tirofiban did not raise the risk of symptomatic intracranial hemorrhage (sICH) or death within 90 days, but the rate of radiological ICH was significantly higher in the tirofiban group than in controls [3]. Other meta‐analyses reported that tirofiban besides not improving functional outcome, raised the occurrence of fatal ICH especially via intra‐arterial administration [4], or found that tirofiban reduced mortality with no increase of sICH or any ICH [2, 5].

Recent studies have drawn attention to the variable efficacy of tirofiban in different clinical scenarios. In a pooled analysis of data from the EVT trial (Direct Endovascular Treatment for Large Vessel Occlusion Stroke) and the RESCUE BT trial (Intravenous Tirofiban Before Endovascular Thrombectomy for Acute Ischemic Stroke) and RESCUE BT Trials among patients with intracranial large vessel occlusion within 4.5 h of onset, tirofiban plus endovascular therapy was comparable to alteplase bridging with endovascular therapy regarding the efficacy and safety outcomes [6]. In a large multicenter trial of patients with ischemic stroke without occlusion of large or medium‐sized vessels, the percentage of patients with a score of 0 or 1 on the modified Rankin scale at 90 days was significantly higher with tirofiban than with aspirin, although there was a low but slightly higher incidence of sICH in the tirofiban group [7].

It is plausible that a more precise definition of candidates for tirofiban targeted treatment could yield more homogeneous results of the benefits of this platelet aggregation inhibitor in the acute stroke setting. Interestingly, in the current issue of the journal, Yue and colleagues [8] investigated the efficacy and safety of intravenous tirofiban administered before endovascular treatment in patients with acute ischemic stroke. Based on data of the RESCUE BT trial, the authors carried out a post hoc analysis focused specifically on patients in which optimal large‐vessel recanalization was achieved, exploring the potential role of tirofiban in improving microcirculatory disturbances. Because of distinct pathophysiological mechanisms of large artery atherosclerosis and cardioembolic stroke subtypes, analyses in these two subgroups were also performed. The primary outcome was favorable functional recovery at 90 days (mRS ≤ 2), and safety outcomes included sICH and 90‐day mortality. In the overall population of acute ischemic stroke patients, tirofiban showed no clinical improvement in the primary outcome variable as compared with placebo. However, better outcomes were found in the subgroup of stroke patients with large artery occlusion and NIHSS scores > 13 (adjusted odds ratio 4.671), while no benefit was observed in cardioembolic stroke patients across NIHSS subgroups. These findings suggest that caution should be exercised when using tirofiban in cardioembolic stroke patients.

The authors argue that possibly, thrombi in large vessel occlusion stroke are more stable and tirofiban may improve microcirculation, which is in contrast to cardioembolic patients, with softer cardiac thrombi and poorer collateral circulation, who may experience worsened microcirculatory disturbances and limited recovery after reperfusion [8]. Additionally, the antiplatelet effects of tirofiban may hinder vascular repair, particularly in cardioembolic patients where thrombus instability may further increase the risk of vessel rupture and hemorrhage [8].

In short and notably, the efficacy of tirofiban may differ between these two ischemic stroke subgroups. It is important to emphasize that acute ischemic stroke is a highly heterogeneous entity [9], and in clinical practice, the study of Yue et al. [8] emphasizes the need of the individualized use of tirofiban according to stroke subtype and pre‐stroke neurological impairment in order to optimize outcomes and minimize risks. Open lines of research include the potential enhanced efficacy of tirofiban combined with other antiplatelet or anticoagulant therapies, as well as the assessment of the efficacy and safety of tirofiban in octogenarians, men versus women, or lacunar versus non‐lacunar infarction, all of which are important and challenging subsets of acute ischemic stroke that merit to be evaluated in further studies.

## Author Contributions

**Adrià Arboix:** conceptualization, writing – original draft, methodology, writing – review and editing. **Olga Parra:** conceptualization, methodology, writing – review and editing.

## Conflicts of Interest

The authors declare no conflicts of interest.

## Data Availability

Data sharing is not applicable to this article as no new data were created or analyzed in this study.
