# Correction to “Tirofiban Benefits the Outcome of Stroke Patients With Large Artery Atherosclerosis Achieving Full Reperfusion and High NIHSS Scores”

**DOI:** 10.1111/ene.70210

**Published:** 2025-05-14

Arboix, A. and Parra, O. (2025), Tirofiban Benefits the Outcome of Stroke Patients With Large Artery Atherosclerosis Achieving Full Reperfusion and High NIHSS Scores. Eur J Neurol, 32: e70070. https://doi.org/10.1111/ene.70070.

This article was incorrectly published as an Invited Review. It is an Editorial.

We apologize for this error.

